# Effects of combined treatment with transcranial and peripheral electromagnetic stimulation on performance and pain recovery from delayed onset muscle soreness induced by eccentric exercise in young athletes. A randomized clinical trial

**DOI:** 10.3389/fphys.2023.1267315

**Published:** 2023-10-11

**Authors:** Hugo Keriven, Alberto Sánchez Sierra, Ángel González de-la-Flor, María García-Arrabé, María Bravo-Aguilar, Marta de la Plaza San Frutos, Guillermo Garcia-Perez-de-Sevilla, Jose Francisco Tornero-Aguilera, Vicente Javier Clemente-Suarez, Diego Domínguez-Balmaseda

**Affiliations:** ^1^ Department of Physiotherapy, Faculty of Sports Sciences, Therapeutic Exercise and Fucntional Rehabiltiation Research Group, Universidad Europea de Madrid, Villaviciosa de Odón, Madrid, Spain; ^2^ Faculty of Phisioterapy and Nursing, Universidad de Castilla-La Mancha, Toledo, Spain; ^3^ Toledo Physiotherapy Research Group (GIFTO), Madrid, Spain; ^4^ Research Group on Exercise Therapy and Functional Rehabilitation, Faculty of Health Sciences, Universidad Europea de Madrid, Madrid, Spain; ^5^ Masmicrobiota Group, Faculty of Health Sciences, Universidad Europea de Madrid, Madrid, Spain

**Keywords:** DOMS (delayed onset muscle soreness), paired-associative electromagnetic stimulation, eccentric exercise and muscle damage, recovery, performances, athletes

## Abstract

**Background:** There is a common interest in finding a common consensus in the approach of athletes suffering from DOMS with the aim of accelerating recovery and thereby enhancing performance. The objective of this study was to observe the effects of a paired-associative transcranial and peripheral electromagnetic stimulation on young athletes suffering from DOMS, induced by 1 h of eccentric and plyometric exercises.

**Methods:** Forty-eight young athletes participated in this randomized control trial: 13 were assigned to the peripheral group (P); 12 were in the control group (Cont); 11 were assigned to the transcranial group (T) and 12 were included in the paired-associative group (Comb). The Visual Analogue Scale (VAS) of pain perception and the mechanical Pressure Pain Threshold (PPT) were the tools used to analyze the symptoms of DOMS. On the other hand, the Half Squat (HS) test evaluated with an accelerometer, and the 30 m sprint velocity (30-mSP) test were used to observe the evolution of the sports performance of the lower limbs. All evaluations were performed before and after the eccentric exercise session that caused DOMS, as well as at 24–48, and 72 h afterward.

**Results:** The AS group improved the symptoms of the induced DOMS, since significant positive differences were observed in the VAS and PPT compared to the other groups (*p* < 0.001). In addition, the AS group showed a significant improvement in the HS and the 30-mSP tests (*p* < 0.001). Based on the results a treatment with both peripheral and transcranial electromagnetic stimulation improves recovery and performance in athletes at 72 h, although these data would need to be verified in future research with a larger sample size.

**Conclusion:** Paired-associative electromagnetic stimulation improved DOMS symptomatology, velocity, and sports performance in the lower limbs.

## Introduction

Delayed Onset Muscle Soreness (DOMS) is a common issue in sports practice, and researchers and clinicians are investigating its origin and seeking strategies to alleviate its symptoms. It has been shown through the decades that many mechanisms and substances are involved in the DOMS apparition. Several studies have been conducted to understand the mechanism of late onset muscle pain (Murase et al., 2010; Sonkodi et al., 2020; Sonkodi, 2021). DOMS often occurs during a return to activity or following unusual exercise, causing discomfort that can lead to decreased performance or even an athlete’s withdrawal from participation (Hickey et al., 2014a; Mori et al., 2014; Hayashi et al., 2017). The release of bradykinin by the sweat during sports activities which conduct to increase the glutamate’s levels in the human body and the nerve growth factor are implicate in the sensitization of the nociceptors (Baker, 2005; Murase et al., 2010). That hyperalgesia induced, combined with a recent theory that suggests the DOMS may be caused by axonal compression (Stifani, 2014; Radovanovic et al., 2015; Colón et al., 2017). This theory proposes that peripheral nerves in the neuromuscular spindle experience micro-lesions and compression due to acute muscle compression and glutamate excitotoxicity, leading to muscle fiber damage (Cheung et al., 2003; Nieman et al., 2005). It is also associated with damage to neighbouring tissues, triggering inflammation (Sonkodi, 2021). This new theory consists of two phases: the Damage phase, where force work leads to muscle lengthening and subsequent acute compression and damage to the neuromuscular spindle, and the Symptomatic phase, where activity-induced damage and inflammation result in pain and protective reflex responses, such as stiffness and decreased strength (Sonkodi et al., 2020).

Peripheral electromagnetic stimulation (PES), also known as transcutaneous magnetic stimulation, is a non-invasive method that delivers a rapid pulse of high-intensity electricity and a magnetic field to the peripheral body, unlike transcranial magnetic stimulation (TES), which targets the brain (Barker, 1991). PES depolarizes peripheral nerves similarly to electrical stimulation, generating a symmetrical biphasic low-intensity current (1–150 Hz) within the body (Barker, 1991). PES offers advantages over TENS, including better penetration through tissues, applicability for patients with hypersensitivity or allodynia, and the ability to be applied over clothing (Kanjanapanang and Chang, 2022). Its mechanism of action involves recruiting peripheral afferents that potentially influence brain activation and neuroplasticity. PES triggers proprioceptive afferents when applied to muscles through direct activation of sensorimotor nerve fibers and indirect activation of mechanoreceptors in muscle fibers (Beaulieu and Schneider, 2013; Beaulieu and Schneider, 2015; Meesen et al., 2011; Sdrulla et al., 2015). In contrast, TES has been developed over the years for various purposes. Lefaucheur et al. (2017) analysed TES parameters and reported its safe use in therapy, particularly for subjects with neurological pathologies or fibromyalgia, where central and peripheral fatigue are significant factors (Lefaucheur et al., 2017). Recent studies have explored the effects of TES on relaxing fatigued muscles after eccentric contractions that lead to DOMS (Celnik et al., 2009; Alder et al., 2019; Delaval et al., 2022). Depending on the targeted area, TES may facilitate muscle relaxation by inhibiting muscle excitation (Vernillo et al., 2021). Additionally, exercise-induced fatigue could be mitigated by combining TES and PES to modulate cortical excitability (Milanović et al., 2011). Therefore, this combined treatment could directly impact the peripheral nervous system of damaged muscles and affect the central nervous system to enhance athlete recovery. This project aims to demonstrate potential performance improvements through muscle recovery by inducing DOMS in young athletes using peripheral and transcranial electromagnetic stimulation.

## Materials and methods

### Study design

A double-blind randomized study was conducted with young athletes, following the protocol and principles of the Declaration of Helsinki [“Declaration of Helsinki of the World Medical Association: Ethical Principles for Medical Research Involving Human Subjects” (World Medical Association, 1991)]. The study adhered to the Consolidated Standards of Reporting Trials (CONSORT) guidelines and was approved by the Research Ethics Committee (reference number: C.I. 23/048-E). The study was also registered (ACTRN12623000677606). A prospective, randomized experimental design with a control group was employed. The study consisted of four groups: the control group (Cont) received no intervention, the Super Inductive (P) group received peripheral electromagnetic stimulation, the Transcranial (T) group received transcranial stimulation, the Combination of Stimulations (Comb) group received a combination of both stimulations. A specific place was used during the whole study for the participants to receive the treatment as belong to one of the group studies. This was made to facilitate the blinding of our study for the participant and the experimenters and make sure that none of them were aware to which group belong any participant. The experimenters assigned to the treatment station in our study, only performed that part and did not communicate with the other members of the research team as the participants.

### Sample size calculation

The sample size was calculated based on an alpha error of 0.5 and a beta error of 0.2 and a standard deviation = 2.61 it was determined a sample of *n* = 22 per group (software G-Power) v3.1). To anticipate a 15% dropout rate during the study, an adjusted sample size was established to 80 participants, divided into four groups (*n* = 20).

### Participants

The study was conducted with students from the European University of Madrid who met the specified inclusion and exclusion criteria. Recruitment of participants was done through flyers, posters, and advertisements in the Faculty of Sport Sciences of the European University of Madrid. The inclusion criteria were as follows: being male between 18 and 35 years of age (i) (Chen et al., 2019), engaging in physical activity at least three times a week for at least 1 year (ii), not having desensitization in the areas treated with peripheral stimulation (iii), not having a diagnosed chronic disease (iv), not having experienced a musculoskeletal injury to the lower limbs in the last 6 months (v), and not being a smoker (vi). Participants with pathologies incompatible with sports practice, those unable to understand the purpose of the sessions, or those who did not meet the inclusion criteria were excluded prior to the start of the study.

### Randomization

Group randomization was performed using the randomization function in Microsoft Office Excel (Microsoft Corporation, Redmond, Washington, United States). Participants were assigned to one of the four study groups as described previously.

### Procedure

**FIGURE 1:**
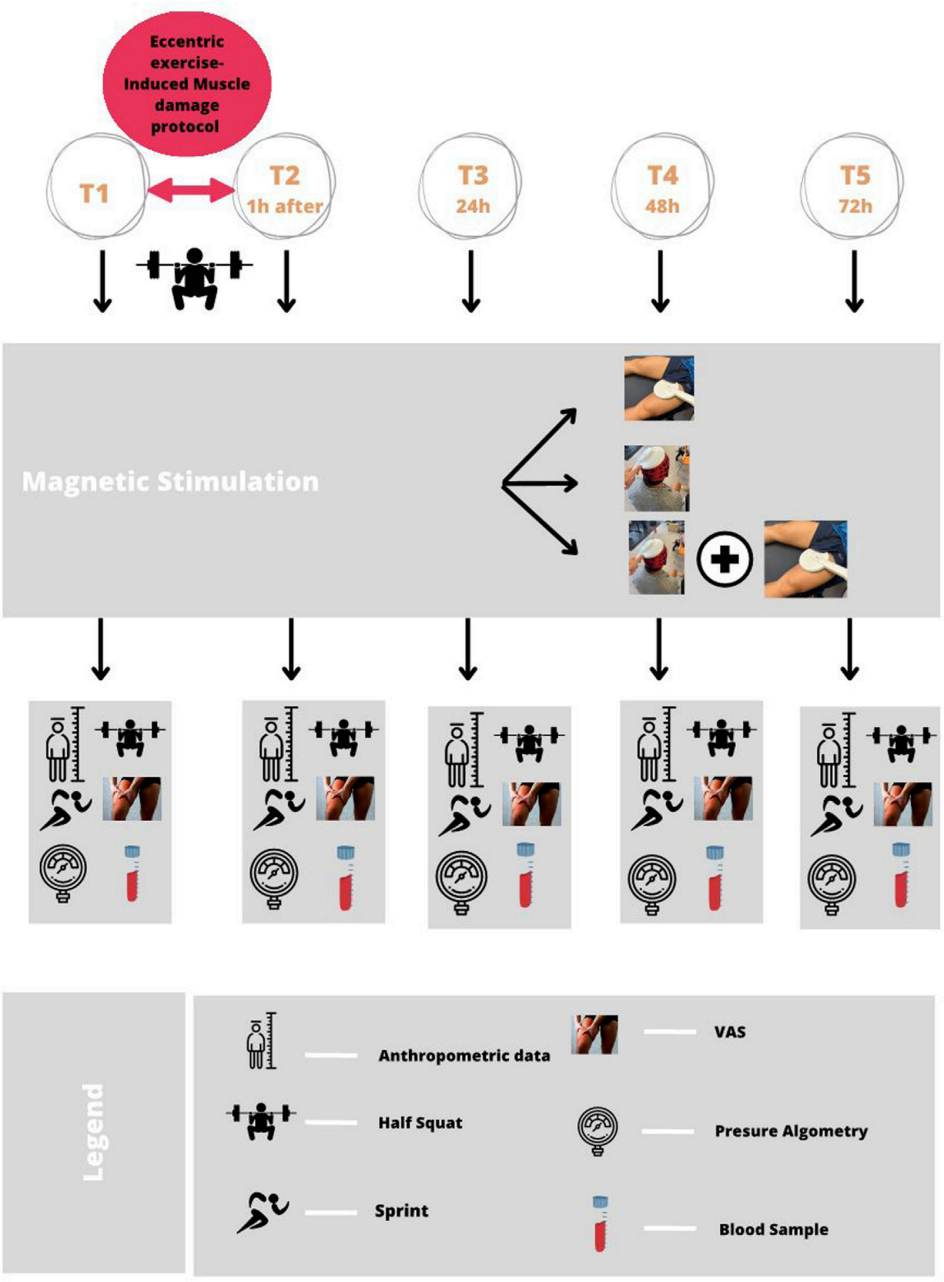
Graphic design of the study and intervention. Pre-post times after 1 h of eccentric exercises as an intervention. Post 24 h, Post 48 h, and Post 72 h: evolution and analysis of the variables during the 24, 48, and 72 h of recovery.

The participants underwent five evaluation sessions as shown in Figure 1. One week before the first evaluation, all participants had a familiarization session. During that session (day one, pre-muscle damage), various measurements were collected, including creatine kinase, blood lactate levels, half squat performance, 30-m speed sprint, pressure pain threshold (PPT) measurement, fatigue perception, and anthropometric data. The same parameters were collected in subsequent sessions, 1 h after, 24, 48, and 72 h after the muscle damage protocol. The impact of the muscle damage protocol was assessed by analysing creatine kinase and blood lactate levels, which were obtained through venous blood samples and analysed using electroenzymatic analysis (Lactate Scout Pro, Musimedic S. L Donostia, Spain). The muscle damage caused by the exercise session was recorded at the beginning and throughout the study to analyse the influence of the treatment on the inflammatory response. Participants were instructed not to engage in physical activities at least 2 days before the study, as enzyme levels could increase following physical activity.

## Intervention

### Eccentric exercise protocol

The session comprised three phases, which involved strength exercises. Firstly, a global warm-up was conducted to prevent injuries. Secondly, the participants performed a series of three intervention exercises, including the Squat exercise with encoder control, using a linear accelerometer to measure 60% of their 1 Repetition Maximum (1 RM). This percentage was calculated according to the protocol developed by González-Badillo et al. (2011), based on the speed at which the subject was able to move the load in meters per second (González-Badillo et al., 2011).

**FIGURE 2:**
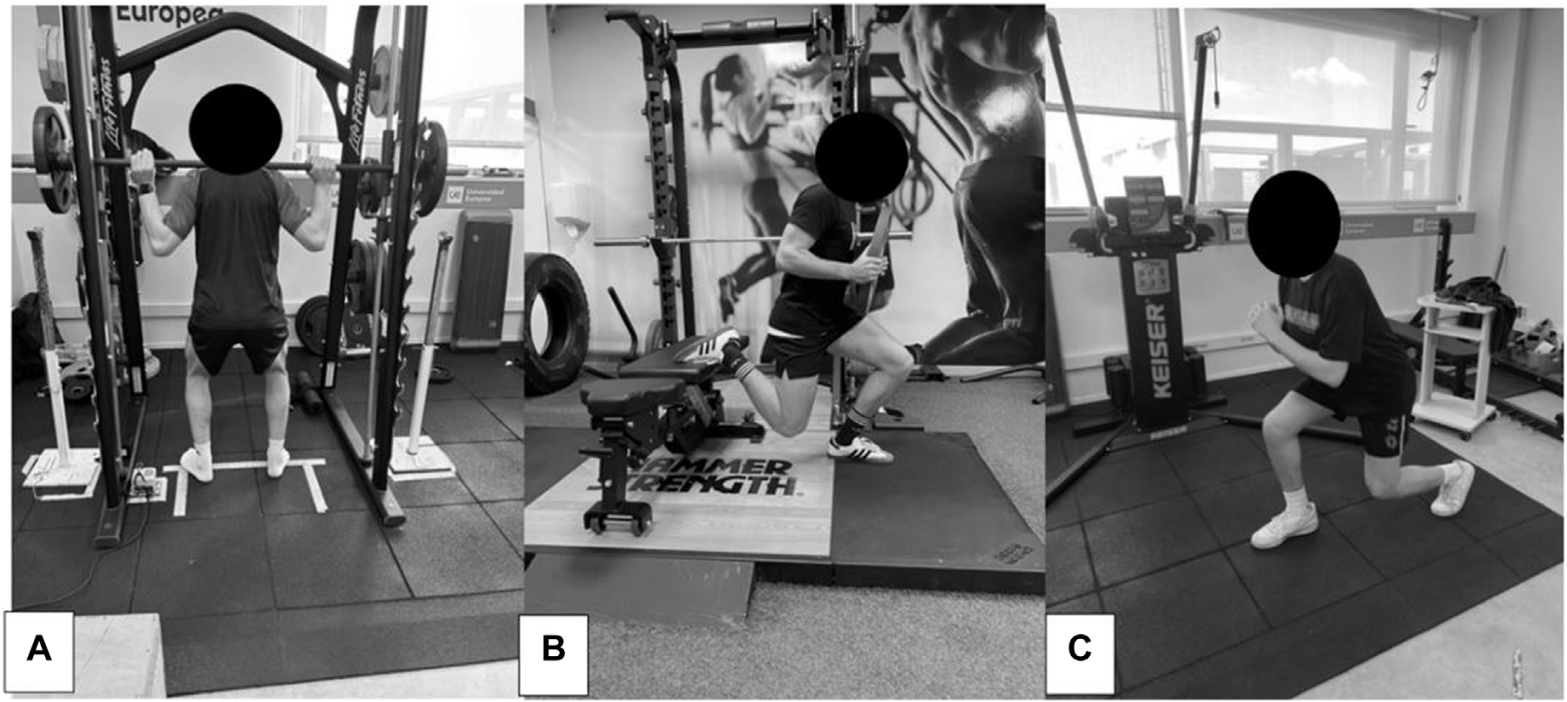
Study subjects performing the eccentric exercise protocol. **(A)**: Front Squat; **(B)**: Bulgarian Squat; **(C)**: Frontal Stride.

The eccentric exercise protocol consisted of the following three exercises:

a) Front Squat: Participants performed 10 sets of 10 repetitions at 60% of their 1 RM, which was calculated during the session prior to the study.
b) Bulgarian Squat: Participants completed 3 sets of 10 repetitions per leg, with the option of adding 5 or 10 kg of additional weight.
c) Front Lunges (Split): Participants also performed 3 sets of 10 repetitions on each leg, with the option of adding 5 and 10 kg of additional weight (Figure 2).

### Peripheral and transcranial electromagnetic stimulation protocol

The participants received different electromagnetic stimulation treatments based on their assigned groups. For the control group (Cont), the machine was placed keeping the coil position in the same place that occupied in the other groups but with the device turned off the machine sound was recorded before the study and play as the same time, in a LTP protocol time which was 5 s stimulation time with 55 s rest between each loud stimulation repeated 5 time to be equal with a classic LTP protocol. In the case of transcranial therapy, it was shown that placing the cap relative to the TES was sufficient to maintain the placebo effect.

Specifically, those in the Super Inductive (P) group received a peripheral electromagnetic stimulation treatment, known as the LTP protocol, consisting of 5 stimulations at 100 Hz each for 5 s, with 55 s of rest between stimulations (Lang et al., 2007; Milanović et al., 2011). The stimulation duration for this group was 10 min.

The Transcranial Stimulation (T) group received a transcranial electromagnetic stimulation treatment of 2000 impulses applied for at least 20 min to the M1 cortex zone (Todd et al., 2003; Dietmann et al., 2023).

The Combined Stimulation (Comb) group received both electromagnetic stimulation therapies, with a stimulation duration of 30 min.

In each group, the treatment began after the eccentric session 1 h after the generated fatigue, in study time this represent at the T2 time. Furthermore, the stimulation time was specific depends on what group was assigned the subject in a range including all study group from 10 min for the SP group to 30 min for the AS group.

**FIGURE 3:**
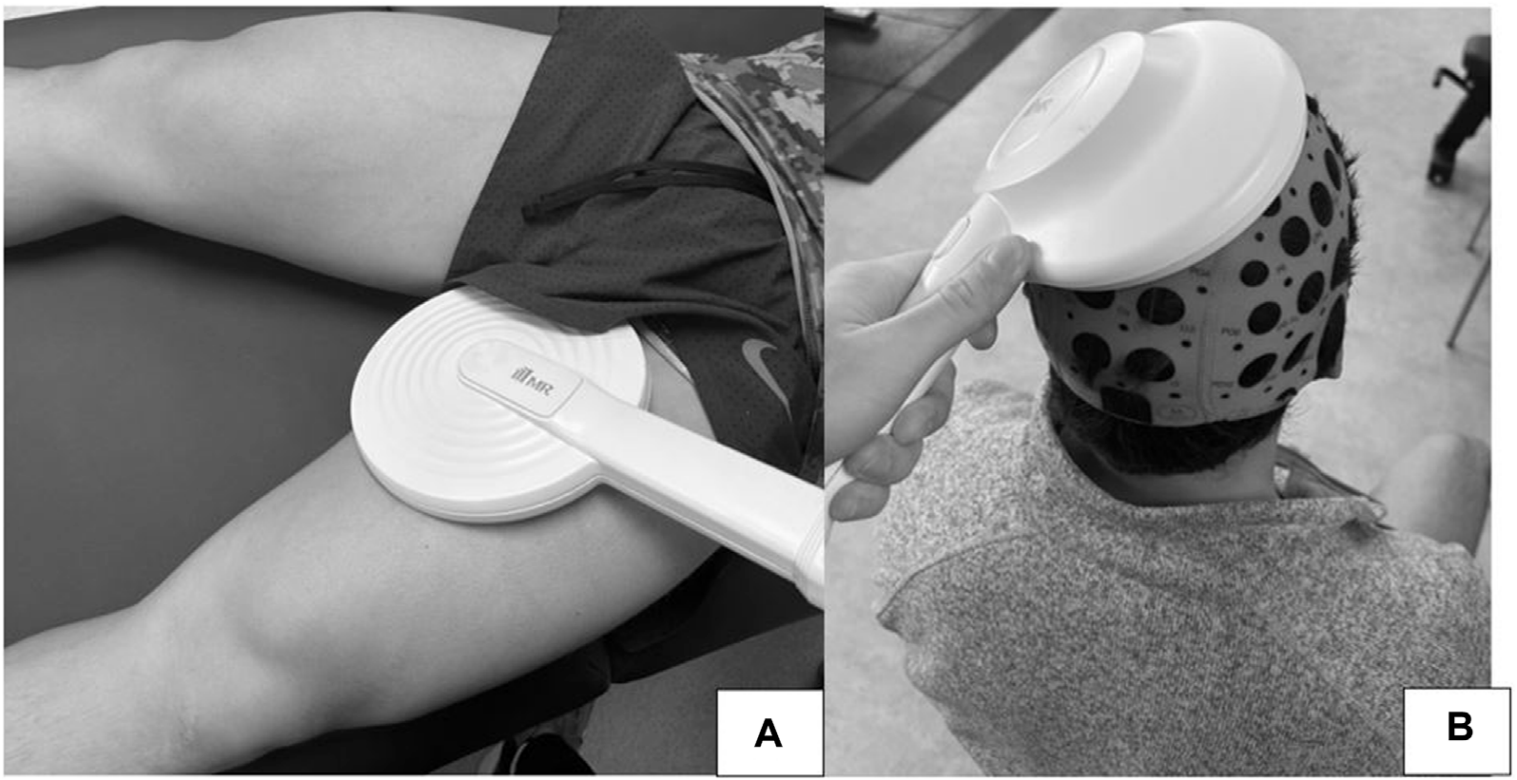
Study subjects treated with peripheral **(A)** and transcranial **(B)** electromagnetic stimulation equipment.

Both transcranial and peripheral electromagnetic stimulation were applied using a MagRex magnetic stimulator with ring shape coil/8 shape coil (MR Inc., Republic of Korea, http://www.mrev.co.kr) (Figure 3).

### Primary outcome measure

#### Half squat

The Half Squat (HS) was recorded as the primary outcome measure in our study, with a load equivalent to 80% of each subject’s body weight. The test was performed using a Smith Multipower machine with a bar guide to prevent anterior-posterior oscillation movements that could interfere with subsequent speed of execution records during the half squat. An encoder (Sensorize C1-P accelerometer) was used to measure the speed of execution in meters/second for each subject (Dominguez-Balmaseda et al., 2020).

### Secondary outcome measures

#### 30 m sprint

Muscle performance was analysed using a 30-m sprint at maximum speed at each time point of the study. Two cells were used to record the time of each run. This measure aims to observe the velocity response and potential changes during the study period. The 30-m sprint is a common velocity race for athletes in both team and individual sports (Pearcey et al., 2015).

### Visual analogue scale—fatigue perception

The Visual Analogue Scale—Fatigue perception (VAS-F) was used to assess fatigue perception, a validated tool to measure the intensity of pain experienced by the subjects. The VAS presents a 100 cm horizontal line, with the extremes representing the extremities of the evaluated symptom: “0” indicating no pain and “100” indicating unbearable pain (Akehurst et al., 2021).

### Pressure pain threshold (PPT)

A mechanical pain threshold analysis was performed using a BASELINE algometer (Wagner instruments, Greenwich, United States). The subjects were assessed bilaterally on a stretcher by a physiotherapist, targeting the vastus medialis. Different points were taken as a reference based on the study by Torres-Chica et al. (2015), Fleckenstein et al. (2017a), and Fleckenstein et al. (2017b). The measure using an algometer for the mechanical pain shown a reliability between experimenters (CCI: 0.64–0.92) y reliability test-retest (CCI: 0.72–0.95) (Tabatabaiee et al., 2020).

### Statistical analysis

SPSS v.25 (IBM, Armonk, NY, United States) was used to conduct the statistical analyses. The data’s normal distribution was assessed using the Shapiro-Wilk test and histograms. Variables with a *p*-value less than <0.05 were considered non-normally distributed, while those with a *p*-value greater than 0.05 were considered normally distributed. To describe the sample, mean and standard deviation were reported for normally distributed variables, while median and interquartile range were reported for non-normally distributed variables. The independent *t*-test or Mann-Whitney *U*-test were used to compare mean values between groups at the baseline for quantitative variables. Homoscedasticity and sphericity were evaluated. When assumptions were met, a 5 × 2 mixed analysis of variance (ANOVA) was conducted, with adjustment for multiple comparisons using the Bonferroni test. A 95% confidence interval was used.

## Results

### Baseline data

**FIGURE 4:**
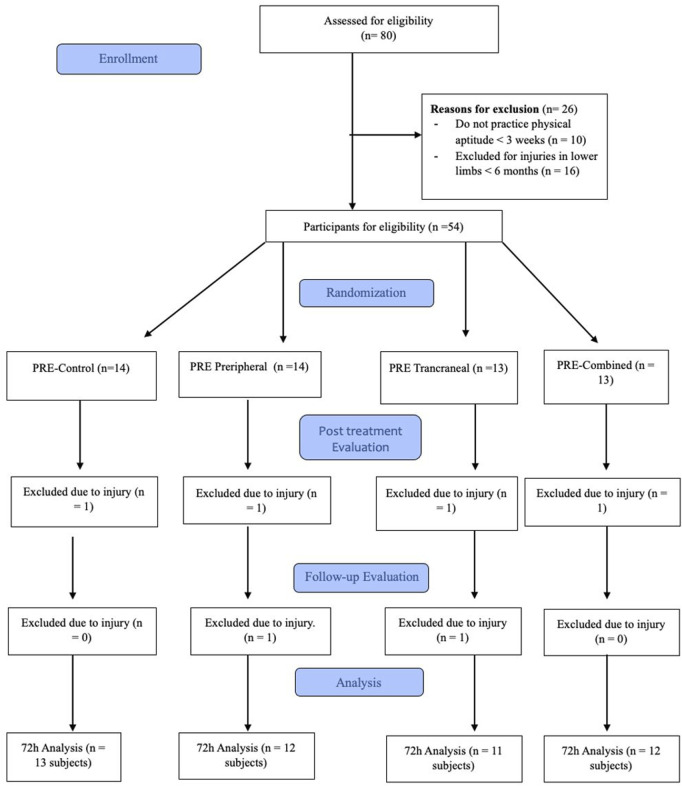
Flowchart of the study following the CONSORT regulations.

A total of 80 volunteers accepted to participate in the study, 26 were excluded before the first evaluation and 4 more were not able to finish the study (Figure 4). Therefore, a total of 48 male participants aged 21.95 ± 4.23 years were included and analysed in the study. The demographic characteristics of the study population did not differ between all individuals in the four groups (Table 1). To corroborate these results, a statistical analysis of the values at the first time point of the study, pre-exercise, was performed, which indicated a homogeneous sample as no correlations were found between the groups at this time point (Table 2). To control the response to the eccentric exercise session was homogenous for each group, the CK and blood lactate levels were monitored.

**TABLE 1:** Descriptive variables of the study population.

Descriptive variables	Total (*n* = 48)	Cont group (*n* = 12)	P group (*n* = 13)	T group (*n* = 11)	Comb group (*n* = 12)	*p*-value
Age (years)	21.95 ± 4.23	21.66 ± 4.24	22.14 ± 5.27	20.54 ± 1.43	23 ± 4.12	0.548
Weight (kg)	74.58 ± 8.94	76.16 ± 8.59	72.61 ± 9.20	71.72 ± 7.17	77.75 ± 8.75	0.311
Size (cm)	179 ± 7.31	181 ± 6.69	176.38 ± 7.07	178.45 ± 4.27	180.33 ± 8.95	0.399
IMC (cm/m^2^)	23.27 ± 2.41	23.27 ± 2.51	23.31 ± 2.43	22.53 ± 2.33	23.89 ± 2.07	0.621

**TABLE 2:** Analysis of the general measures in the pre-exercise (T1).

Variable	Cont (*n* = 12)	P (*n* = 13)	T (*n* = 11)	Comb (*n* = 12)	*p*-value
Lactate (mmol/L)	2.34 ± 3.04	1.38 ± 0.17	2.43 ± 3.50	1.52 ± 0.24	0.560
Creatin Kinase (mmol/L)	66.58 ± 6.88	65.61 ± 6.22	67 ± 7.91	66.25 ± 8.41	0.972
Algometry (PPT) (kg/cm^2^)	10	10	10	10	0.00
Half Squat (m/s)	0.81 ± 0.11	0.86 ± 0.14	0.82 ± 0.13	0.92 ± 0.09	0.125
VAS-F (mm)	23.75 ± 20.67	21.76 ± 19.77	19.45 ± 15.31	21.66 ± 14.66	0.954
30 m Sprint (s)	5.342 ± 0.073	5.287 ± 0.07	5.403 ± 0.076	5.134 ± 0.073	0.961

**FIGURE 5:**
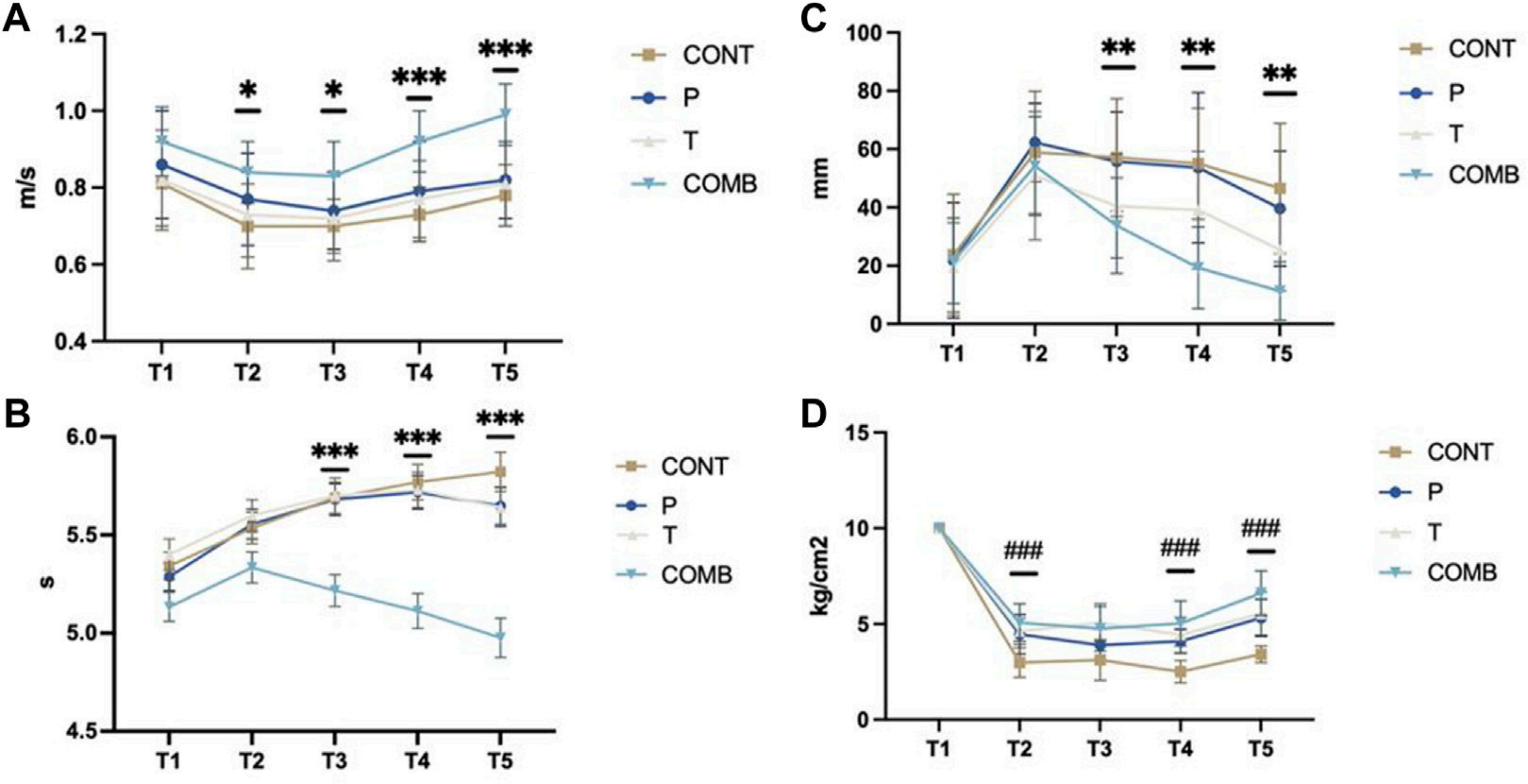
Main results of the study. Describe as; **(A)**: Half Squat; **(B)**: 30-m Sprint; **(C)**: VAS-F; **(D)**: Pressure Algometry (PPT).

An increased in the CK was observed at 1, 24, 48, and 72 h after the eccentric session in the four groups (*p* < 0.001) and no interaction between group was detected in pre (*p* = 0.972), post 1 h (*p* = 0.362), post 24 h (*p* = 0.103), post 48 h (*p* = 0.105) and post 72 h (*p* = 0.616). An increase in blood lactate levels was observed 1 h post eccentric session in all the groups (*p* < 0.001) and no interaction between group was observed (*p* = 0.296) (Figure 5). Baseline measures of the dependent variables did not show significant differences between groups (Table 2).

### Primary outcome

Group-by-time interaction was observed for HS (*p* < 0.001) (Table 3). From 1 h post to 24 h the Comb group showed an increase of the HS speed from the Cont group (0.132 ± 0.046; *p* = 0.004 and 0.12 ± 0.04; *p* = 0.015). From 48 to 72 h the Comb group showed an increase of the HS speed compared to P group (0.126 ± 0.041; *p* = 0.021 and 0.16 ± 0.03; *p* = 0.021); the Cont group (0.18 ± 0.04; *p* < 0.001 and 0.2 ± 0.04; *p* < 0.001) and finally with the T group (0.14 ± 0.04; *p* = 0.009 and 0.175 ± 0.041; *p* < 0.001).

**TABLE 3:** Differences between the groups study for the dependent variables.

	T1 (baseline)	T2 (post 1 h)	T3 (post 24 h)	T4 (post 48 h)	T5 (post 72 h)	F	P (time × group)	*η* ^2^p
	Primary Outcome							
	Half Squat (m/s)							
Cont (*n* = 12)	0.81 ± 0.11	0.7 ± 0.11^a^	0.7 ± 0.07^a^	0.73 ± 0.07^a^	0.78 ± 0.08^a^	7.917	<0.001	0.351
P (*n* = 13)	0.86 ± 0.14	0.77 ± 0.12	0.74 ± 0.1	0.79 ± 0.13^a^	0.82 ± 0.1^a^			
T (*n* = 11)	0.82 ± 0.13	0.73 ± 0.11	0.72 ± 0.11	0.77 ± 0.1^a^	0.81 ± 0.11^a^			
Comb (*n* = 12)	0.92 ± 0.09	0.84 ± 0.08	0.83 ± 0.09	0.92 ± 0.08	0.99 ± 0.08			
	Secondary Outcome							
	30-m sprint (s)							
Cont (*n* = 12)	5.342 ± 0.073	5.535 ± 0.079	5.69 ± 0.084^a^	5.773 ± 0.089^a^	5.822 ± 0.1^a^	19.231	<0.001	0.567
P (*n* = 13)	5.287 ± 0.07	5.555 ± 0.076	5.681 ± 0.081^a^	5.718 ± 0.086^a^	5.648 ± 0.096^a^			
T (*n* = 11)	5.403 ± 0.076	5.606 ± 0.082	5.711 ± 0.088^a^	5.726 ± 0.093^a^	5.64 ± 0.104^a^			
Comb (*n* = 12)	5.134 ± 0.073	5.334 ± 0.079	5.217 ± 0.084	5.113 ± 0.089	4.977 ± 0.1			
	VAS-F (mm)							
Cont (*n* = 12)	23.75 ± 20.67	58.91 ± 20.98	57.08 ± 20.16^a^	55 ± 19.06^a^	46.5 ± 22.29^a^	10.204	<0.001	0.41
P (*n* = 13)	21.76 ± 19.77	62.3 ± 13.44	55.69 ± 17.06^a^	53.61 ± 25.75^a^	39.61 ± 19.73^a^			
T (*n* = 11)	19.45 ± 15.31	50.9 ± 22	40.45 ± 17.81	39.09 ± 20.1	25.45 ± 14.04			
Comb (*n* = 12)	21.66 ± 14.66	54.16 ± 16.89	33.75 ± 16.39	19.33 ± 14.04	11.25 ± 10.02			
	PPT (kg/cm^2^)							
Cont (*n* = 12)	10	2.98 ± 0.76^a,b,c^	3.12 ± 1.06	2.51 ± 0.58^a,b,c^	3.42 ± 0.44^a,b,c^	13.945	<0.001	0.487
P (*n* = 13)	10	4.46 ± 1.03	3.89 ± 0.84	4.1 ± 0.63	5.32 ± 0.97^a^			
T (*n* = 11)	10	4.62 ± 0.69	5.07 ± 10.98	4.43 ± 0.93	5.54 ± 1.12			
Comb (*n* = 12)	10	5.07 ± 0.98	4.75 ± 1.17	5.03 ± 1.18	6.61 ± 1.16			

P, super inductive group; Cont, control group; T, transcranial group; Comb, combined group; VAS, visual analogic scale to evaluate fatigue; PPT, pressure pain threshold; *η* ^2^p, partial eta squared; a, interaction with the AS group (*p* < 0.001); b, interaction with the SP group (*p* < 0.001); c, interaction with the TS group (*p* < 0.001).

### Secondary outcome

Group-by-time interaction was observed for the 30 m sprint (*p* < 0.001) (Table 3). Between 24 and 72 h, the Comb group showed a significant reduction in sprint time compared to the other study groups (*p* < 0.001). At 24 h post-exercise, the Comb group demonstrated a significantly lower sprint time compared to the Cont group (0.473 ± 0.119; *p* = 0.002), the P group (0.463 ± 0.117; *p* = 0.002), and the T group (0.61 ± 0.12; *p* < 0.001). At 48 h post-exercise, similar interactions were observed with the Cont group (0.65 ± 0.12; *p* < 0.001), the P group (0.60 ± 0.12; *p* < 0.001), and the T group (0.61 ± 0.12; *p* < 0.001). At 72 h post-exercise, the same interaction was evident with the Cont group (0.84 ± 0.14; *p* < 0.001), the P group (0.67 ± 0.13; *p* < 0.001), and the T group (0.66 ± 0.14; *p* < 0.001).

Additionally, a group-by-time interaction was analysed for VAS-F (Visual Analogue Scale—Fatigue perception) (*p* < 0.001) (Table 3). At 24 h post-exercise, the first interaction was observed between the Comb group and the Cont group (23.33 ± 7.3; *p* = 0.016), and the Comb group with the super inductive group (21.94 ± 7.16; *p* = 0.022). At 48 h post-exercise, similar interactions were observed between the Comb and Cont group (35.66 ± 8.29; *p* = 0.001), and the Comb and P group (34.28 ± 8.13; *p* = 0.001). Finally, at 72 h post-exercise, an interaction was observed between the Comb and Cont group (35.25 ± 7.45; *p* < 0.001), and between the Comb and P group (28.36 ± 7.3; *p* = 0.002). No interaction was observed between the T and Comb groups during the study time.

Similarly, a group-by-time interaction was found for PPT (Pressure Pain Threshold) between groups (*p* < 0.001) (Table 3). At 1-h post-session, interactions were observed between the P and Cont group (1.48 ± 0.35; *p* = 0.001), the T and Cont group (1.64 ± 0.37; *p* < 0.001), and the Comb and Cont group (2.09 ± 0.36; *p* < 0.001). At 48 h post-session, interactions were observed between the P and Cont group (1.59 ± 0.34; *p* < 0.001), the T and Cont group (1.92 ± 0.36; *p* < 0.001), and the Comb and Cont group (2.51 ± 0.35; *p* < 0.001). At 72 h post-session, interactions were observed in the Comb group and the P group (1.29 ± 0.38; *p* = 0.01), and the Comb group and the Cont group (3.19 ± 0.39; *p* < 0.001). Additionally, interactions were observed between the P and Cont group (1.89 ± 0.38; *p* < 0.001), and between the T and Cont group (2.12 ± 0.4; *p* < 0.001).

Furthermore, a time effect was observed for HS, 30 m sprint, VAS-F, and PPT (*p* < 0.001). Despite the recovery of maximal strength after the muscle damage protocol, HS speed, sprint velocity, VAS-F, and PPT did not return to their baseline values and increased in the association group (Table 3).

## Discussion

The present study aimed to investigate the influence of a combination of transcranial and peripheral electromagnetic stimulation on the recovery and performance of young athletes suffering from Delayed Onset Muscle Soreness (DOMS) in the quadriceps. The combination of both stimulations was hypothesized to improve Half Squat (HS) speed, sprint velocity, Visual Analogue Scale—Fatigue perception (VAS-F), and Pressure Pain Threshold (PPT) in the quadriceps.

Eccentric exercise sessions, which induce DOMS, are commonly included in training dynamics to improve performance. Various research teams have explored strategies to counteract DOMS, such as the use of Transcutaneous Electrical Nerve Stimulation (TENS) or cryotherapy. In this study, we aimed to analyse the effects of transcranial electromagnetic stimulation targeting the M1 area of the brain, involved in motor tasks and central fatigue, combined with peripheral electromagnetic stimulation applying the LTP protocol through a superinductive device in male athletes with induced DOMS (Todd et al., 2003; Dietmann et al., 2023).

The study considered the necessary recovery time after high-intensity efforts, with at least 72 h recommended for a complete return to the baseline state. We chose to analyse performance at 72 h after inducing DOMS.

**FIGURE 6:**
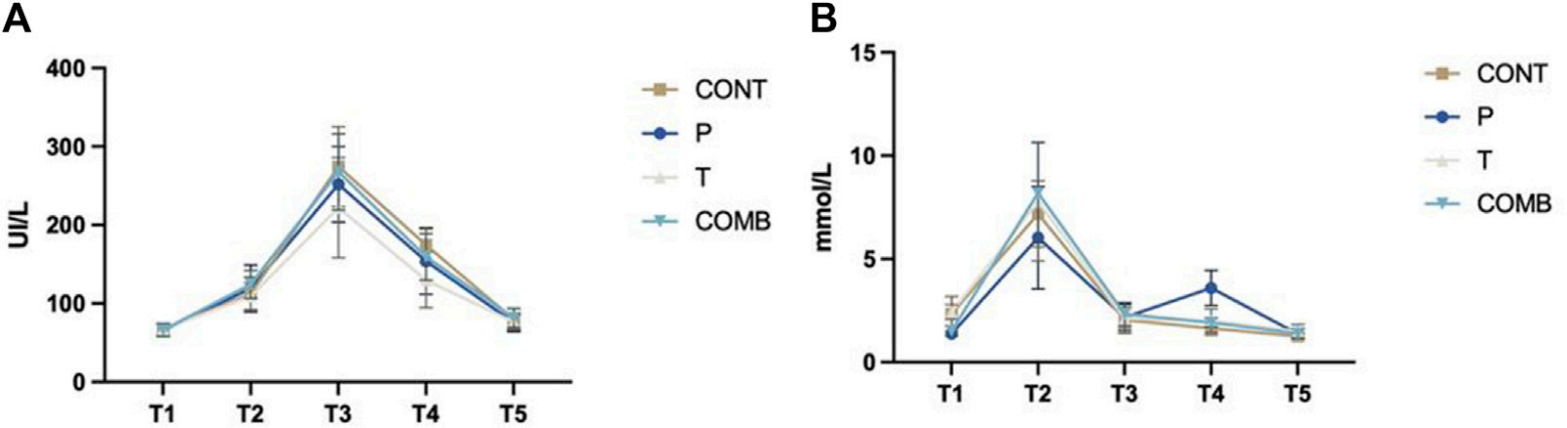
Blood lactate **(A)** and Creatine Kinase **(B)** concentration levels after exercise-induced delayed onset muscle soreness comparing the study’s groups.

First, no interaction was observed between groups for the CK and Lactate concentration (Figure 6). Expressly, there was no difference in muscle damage, which indicate that all the subjects were affected by the eccentric session regardless their baseline statue (Table 3). Nevertheless, the performances of the lower body showed differences between groups early after the eccentric session which could indicate that acute stimulation central and peripheral could struggle the prompt decrease of the performance, that is the purpose of the following analyse in based on our study results.

Lower body performance measured through HS showed significant differences between groups, with better recovery in the Comb group where both stimulations were applied. These findings are in line with Domínguez-Balmaseda et al. (2020) who studied post-eccentric exercise session recovery in young athletes up to 72 h later (Dominguez-Balmaseda et al., 2020). However, the results obtained in our study differ from the study carried out by Keriven et al. (2023), possibly due to a smaller population and the use of only peripheral stimulation in their study (Keriven et al., 2023). The combination of transcranial and peripheral stimulation in our study showed improved execution speed of the half squat movement due to the synergistic effects on the excitability of the central and peripheral nervous systems. Transcranial stimulation modulates the excitability of the primary motor cortex, involved in the planning and execution of movement, while peripheral stimulation directly activates muscle fibers, resulting in a faster and more powerful muscle response. The simultaneous activation of the central and peripheral nervous systems can lead to greater efficiency in force generation and movement execution (Matsuo et al., 2022; Qin et al., 2023).

Regarding the 30 m sprint, our results resemble those of Pearcey et al. (2015), who observed an improvement in running time after inducing DOMS (Pearcey et al., 2015). This suggests that peripheral treatment can reduce the impact of compressions exerted by damaged muscles on peripheral nerve endings. In contrast, Kritikos et al. (2021) obtained different results in a study after inducing DOMS using supplementation, highlighting the potential differences between treatments (Kritikos et al., 2021). As shown in our study results (Figure 5), in all groups the sprint performance reduced at the T2 time versus the baseline which could traduced as DOMS’s impact by a modification of the ability to realize the motor task for the subjects. After the eccentric session, every group suffered an increase between 0.2 and 0.4 s of their 30-m sprint time (Table 3), that can be express as a reduction of the performance and clearly related with the DOMS. However, following the analysis of the sprint performance an evident impact of the combined treatment would be identified in the Comb group results (Table 3; Figure 5). As shown graphically and by the marks, the subjects not only recovered from the DOMS but improve their time traduced by a time interaction between the T5 time (post 72 h) and the baseline (Table 3). Our study supports the fact that a combination of transcranial and peripheral electromagnetic stimulation can improve lower body muscular performance by stimulating the M1 zone for motor skill learning and directly stimulating the micro-injury zone, enhancing motor task performance.

Pain perception measured by the VAS-F scale showed significant differences between groups. Suggested that the treatment involved in our present study participated to reduce the fatigue and pain sensation in the Comb group especially in regards with the Cont group as show in the Table 3. That might be due to the combine effects of both stimulation which add a distal and central inhibition who can conduct to a decrease of fatigue perception in our patient. However, Fan and Sdrulla (2020) demonstrated that distal hypoalgesia developed by athletes does not always fully represent the demands of sport (Fan and Sdrulla, 2020). Our study differs from Guinot et al. (2021) who found that transcranial stimulation alone in patients suffering from fibromyalgia was not effective in reducing pain perception. The differences in patient characteristics and the addition of peripheral stimulation in our study might explain these contrasting results (Guinot et al., 2021).

Mechanical pain analysis using pressure algometry showed significant differences between groups, indicating that combining both stimulations might lead to these results. Furthermore, the early differences between groups 1 h after the eccentric session suggested early therapeutics effects especially in the Comb group. Those differences were observed as well in the posterior times which indicate a continue effect and contribute to reduce the distal hypoalgesia by the peripheral sensibilization and the central sensibilization. Otherwise, the device and protocol used in this study were based on previous one Fleckenstein et al. (2017a), so a baseline measure was realised to make sure that the subjects were not suffering DOMS before the study that could explain the results at T1 time in the Table 3 (Fleckenstein et al., 2017a). Similar findings were observed by Stefanelli et al. (2019) in their study on distal hypoalgesia during DOMS recovery, suggesting that DOMS is a combination of both central and peripheral fatigue (Stefanelli et al., 2019). The significant differences in the PPT were observed from 24 to 72 h post DOMS, where a lower threshold is usually observed due to the natural recovery process.

## Limitations

There are several limitations to consider in the present study. Firstly, it is important to note that the findings found here may not apply to female or less active population. To obtain a more comprehensive understanding, it would be advantageous to investigate in future studies the effectiveness of our treatment in diverse population, such as including female participants or individuals with lower levels of physical activity.

In future studies, it would be valuable to include parameters such as reactivity tests or the triple hop test, as these are essential for assessing change-of-direction capacity in many sports. Additionally, evaluating electromyography (EMG) parameters could provide insights into peak torque in the quadriceps and MEP (motor evoked potential) to analyse both peripheral and central fatigue. Examining muscle recruitment with the combined treatment could also be relevant (Uehara et al., 2022).

Interacting with athletes of similar competitive levels or with the same sports specialization could be beneficial in future research to assess the impact of this treatment at the beginning of a soccer team season or during the early stages of return to training, particularly in terms of injury prevention and recovery (Hickey et al., 2014b; Clemente et al., 2021).

Incorporating the *t*-test, which measures change-of-direction speed, would provide further insights into the influence of transcranial and peripheral electromagnetic stimulation on physical performance (Pearcey et al., 2015).

## Lines for future research

Based on the positive results observed between groups in the present study regarding strength and pain sensation in the quadriceps, future studies with larger sample sizes or comparisons against traditional DOMS recovery methods should be conducted to further investigate the effects of combining transcranial and peripheral electromagnetic stimulation on DOMS-associated symptoms and performance.

In upcoming studies, parameters such as heart rate variability or surface electromyography should be evaluated, given their previously demonstrated involvement in post-exertional recovery.

Finally, it would be of interest to test the efficacy of our treatment in other populations, such as women or individuals with lower physical activity levels, to explore its broader applicability and potential benefits.

## Conclusion

The combination of transcranial and peripheral electromagnetic stimulation appears to have positive effects on recovery and performance in young athletes suffering from DOMS. These findings suggest that the simultaneous activation of the central and peripheral nervous systems can lead to improved muscle performance and pain perception reduction. Future studies may further explore the mechanisms underlying the combined effects of these stimulation techniques and their potential applications in different populations and athletic scenarios.

## Data Availability

The datasets presented in this study can be found in online repositories. The names of the repository/repositories and accession number(s) can be found in the article/Supplementary Material.
